# Perceptual Tuning Influences Rule Generalization: Testing Humans With Monkey-Tailored Stimuli

**DOI:** 10.1177/2041669519846135

**Published:** 2019-04-29

**Authors:** Andrea Ravignani, Piera Filippi, W. Tecumseh Fitch

**Affiliations:** Department of Cognitive Biology, Faculty of Life Sciences, University of Vienna, Vienna, Austria

**Keywords:** artificial grammar learning, generalization, abstraction, statistical learning, conspicuousness, pattern perception

## Abstract

Comparative research investigating how nonhuman animals generalize patterns of auditory stimuli often uses sequences of human speech syllables and reports limited generalization abilities in animals. Here, we reverse this logic, testing humans with stimulus sequences tailored to squirrel monkeys. When test stimuli are familiar (human voices), humans succeed in two types of generalization. However, when the same structural rule is instantiated over unfamiliar but perceivable sounds within squirrel monkeys’ optimal hearing frequency range, human participants master only one type of generalization. These findings have methodological implications for the design of comparative experiments, which should be fair towards all tested species’ proclivities and limitations.

Several comparative studies on rule learning have tested nonhuman mammals and birds using stimuli of debatable ecological validity, such as human-spoken nonsense syllables (e.g., Fitch & Hauser, 2004, see also reviews in Fitch & Friederici, 2012; ten Cate & Okanoya, 2012; Wilson et al., 2019). Such studies report some pattern learning abilities, punctuated by failures. These failures raise the question: Do animals have limited pattern generalization abilities, or could limited perceptual appropriateness of test stimuli hinder generalization? It would obviously be nonsensical to use ultraviolet stimuli to test a species lacking ultraviolet vision. But might colored stimuli designed by a tetrachromatic avian experimenter lead to poor generalization by trichromatic human participants, despite being visible and discriminable to them? In other words, should we be fairer to animals when testing their generalization abilities?

Species-tailored stimuli can trigger or favor pattern generalization in nonhuman primates (Ravignani, Sonnweber, Stobbe, & Fitch, 2013; Ravignani & Sonnweber, 2017; Reber et al., 2019). However, to our knowledge, no previous pattern-learning study has varied perceptual conspicuousness of the experimental stimuli, to evaluate the effect of perceptual tuning on pattern processing across multiple species. Here, we tested how perceptual conspicuousness and sensory familiarity affect rule generalization by human participants.

To do so, our study included two experimental conditions: the *conspecific stimuli condition* (hereafter CSC), which included nonsense strings of spoken consonant-vowel syllables, and the *heterospecific stimuli condition* (hereafter HSC), which included artificial strings made of sine wave tones *tuned* to squirrel monkeys’ hearing frequency range (see Supplementary Material). Syllables in the CSC were divided in two easily discriminable classes of sounds, spoken by either a male or a female speaker. Tones in the HSC also occupied two frequency ranges: Tones varied around a mean frequency of 2 kHz (*lower tones*) or around a mean frequency of 11 kHz (*higher tones*). Notably, the heterospecific stimuli, being designed to suit squirrel monkeys, were less familiar and perceptually conspicuous to humans than the conspecific ones. Previous studies on squirrel monkeys, marmosets, and chimpanzees, using the same (Ravignani et al., 2013; Reber et al., 2019) or similar (Ravignani & Sonnweber, 2017) stimuli to those used here, that is, specifically tailored to these species’ hearing range, showed successful pattern generalization.

Stimuli were designed to assess the effect of these two conditions on humans’ ability to generalize an AB^n^A pattern (ABA, ABBA, ABBBA, etc.). Specifically, within each condition, participants were habituated to stimuli created as follows: In the CSC, one syllable spoken by the male speaker occurred in first and last positions of the sequence, and one to three syllables spoken by the female speaker occurred in the middle; similarly, in the HSC, one lower tone occurred in first and last positions of the sequence, and one to three higher tones occurred in the middle (Ravignani et al., 2013). Thus, male voice and lower tones represented *lower pitched stimuli* (class L) and female and higher tones represented *higher pitched stimuli* (class H), and in each condition, habituation sequences were LHL, LHHL, and LHHHL (see Table A1 in Supplementary Material).

Prior to the experiment start, participants performed an audiometric test measuring their ability to hear tones in the frequency range of the HSC: Only successful participants were included in the analysis of pattern rule extraction and generalization abilities investigated here (see Supplementary Material).

The experiment started with a habituation phase that included 36 stimuli presented in different randomized orders across participants. This was followed by two tests (of 16 trials each, 8 of which featured stimuli following the same pattern as the habituation stimuli) where participants had to rate new sequences of sounds as *similar to* or *different from* those heard in the habituation phase: (a) generalization of the same pattern to novel, longer sequences including more H units in the middle and units of similar frequencies as in the habituation (*length or category generalization*) and (b) generalization over the more abstract AB^n^A pattern, where the position of high-pitched and low-pitched stimuli was swapped compared with the stimuli used in the habituation phase (*structural generalization*). Hence, in the structural generalization test, participants had to classify HL^n^H patterns as instances of the same structural rule as the LH^n^L pattern heard in the habituation phase. Across all test phases, order of trials was randomized across participants. Instructions were minimized to make this work comparable with similar experiments on nonhuman animals.

Four participants performed at chance on the audiometric test and were thus excluded from further analyses. For each individual and condition, we computed the probability of judging a test stimulus’ pattern as *similar* or *different* to the pattern extracted in the habituation phase (Figure 1). The number of individuals succeeding in the length generalization test (Figure 1, left side) is identical in CSC and HSC: 10 participants performed above chance (one-tailed binomial test, *p* < .05). In the structural generalization test, six participants were above chance in CSC (top-right), while only three participants achieved significance in HSC (bottom-right). A binomial logit GLMM provided similar results: Stimulus pattern (consistent vs. inconsistent with the habituation pattern), generalization type (length or category vs. structural), and experimental condition (CSC vs. HSC) all significantly affected participants’ responses in the comparable direction (Table 1).

**Figure 1.**
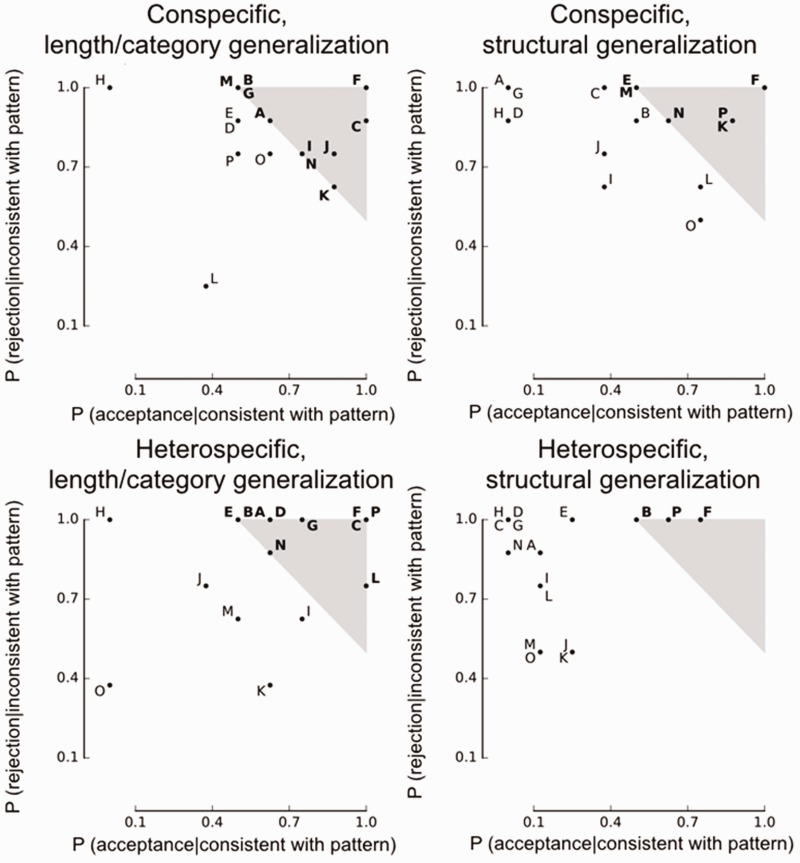
Estimated probabilities of correct responses for each condition (the four graphs) and participant (denoted by a capital letter). Above-chance performance binomial *p* < .05 (shaded grey area and bold font) could be achieved by correctly accepting pattern-consistent stimuli (high abscissa) or rejecting pattern-inconsistent stimuli (high ordinate).

**Table 1.** Results of the Model Estimation for Significant Effects.

Dependent variable (similar vs. different)	Estimate	*SE*	*z*	*p*
Intercept	−1.2362	0.4828	−2.560	<.05
Pattern	2.6452	0.6985	3.787	<.01
Generalization type	0.7031	0.2727	2.579	<.01
Experimental condition	−1.3865	0.6294	−2.203	<.05
Generalization Type × Experimental Condition	1.3865	0.4062	3.413	<.01
Pattern × Generalization Type × Experimental Condition	−1.7569	0.6402	−2.744	<.01

*Note.* α = .05; all *p* values two-tailed. Interactions Pattern × Generalization Type and Pattern × Experimental Condition (not shown) were not significant, all |*z*| < 1.88, all *p* > .05. Crucially, all main effects and only those interaction terms containing “generalization type” and “experimental condition” significantly affected participants’ responses.

Cross-species pattern learning experiments test animals’ ability to generalize an acquired pattern over a range of stimuli that differ in types of generalizations (Kriengwatana, Spierings, & ten Cate, 2015) but often neglect the species-specific perceptual tuning of test stimuli (Fitch & Hauser, 2004; Fitch & Friederici, 2012; ten Cate & Okanoya, 2012; Wilson et al., 2019). Our results illustrate the importance of stimulus audibility and perceptual familiarity in this research paradigm. Strikingly, a considerable portion (20%) of our participants were at chance on an audiometry test of high-frequency sounds in discriminating between tones used to build test sequences adapted for a nonhuman primate species, and those who succeeded were still less likely to succeed at pattern extraction in the structural generalization test in the heterospecifically tuned condition.

Nonhuman animals’ capacity for pattern generalization may, we conclude, be affected by stimuli’s perceptual conspicuousness to the tested individuals (Kriengwatana et al., 2015). Our results indicate that limitations in perceptual relevance or audibility of stimuli may lead to failure to generalize over complex patterns. Increasing the comparative methodological fairness of stimuli will thus enhance accuracy in cross-species investigations of cognition.

## Supplemental Material

Supplemental material for Perceptual Tuning Influences Rule Generalization: Testing Humans With Monkey-Tailored Stimuli Click here for additional data file. Supplemental Material for Perceptual Tuning Influences Rule Generalization: Testing Humans With Monkey-Tailored Stimuli by Andrea Ravignani, Piera Filippi and W. Tecumseh Fitch in i-Perception
