# Supplementary material for: Perceptual Tuning Influences Rule Generalization: Testing Humans With Monkey-Tailored Stimuli
Source: Iperception. 2019 Apr 29;10(2):2041669519846135. doi: 10.1177/2041669519846135 (PMC6488782; doi:10.1177/2041669519846135)
Supplement: Supplemental material for Perceptual Tuning Influences Rule Generalization: Testing Humans With Monkey-Tailored Stimuli [file Supplemental_Material.pdf]

Perceptual tuning influences rule generalization: Testing humans with monkey-tailored stimuli

Supplementary materials

Andrea Ravignani, Piera Filippi, W. Tecumseh Fitch

Extended Methods

Stimuli

The stimuli were originally designed to assess perception and generalization of the rule AB^nA, indicating any pattern having one A at the beginning, one A at the end, and any number of Bs in between (Ravignani et al, 2013; Sonnweber et al., 2015; Ravignani & Sonnweber, 2017; Reber et al., 2019).

A and B symbols represented categories of tonal stimuli, each of which could be mapped to a range of low-pitched (L) or high-pitched (H) sound tokens. Auditory patterns were generated by concatenating tokens, which were either pure sine wave tones adapted to monkeys' hearing (heterospecific phase) or human nonsense syllables (conspecific phase). Each category (L and H) consisted of 44 stimuli. Frequencies of the heterospecific stimuli were randomly, uniformly sampled from an interval centred at 2 kHz (mean frequency of stimuli in category L) and 11 kHz (category H). In both categories, the endpoints of the intervals were located at +- 10% of the mean. Although both sets of frequencies were audible and discriminable to all of our subjects, the high ones were outside of the normal range of musical pitches (about 5 kHz – the highest note on a piano is about 4200 Hz), and thus not in a range most humans spend much time listening to or categorizing. The duration of each tone was randomly sampled between 210 and 240 sec. Tones were concatenated using trapezoidal envelopes of 250 sec each, granting equal durations to stimuli with the same number of tones.

For the conspecific stimuli, the L category consisted of lower-pitch nonsense syllables, uttered by an adult human male, and the H category consisted of higher-pitch syllables, uttered by a human female. Hence conspecific stimuli were strings of randomly-sampled nonsense syllables with no meaning attached: all that mattered was the voice category of each token, male vs. female.

Participants and Experimental procedure

Twenty participants (11 female, Median age: 24.5), recruited at the University of Vienna, were individually tested in a quiet laboratory. Stimuli were played binaurally over headphones. Although human hearing range is commonly given as 20 Hz – 20 kHz, in reality thresholds increase considerably above 15 kHz, and most older adults show progressive loss of high-frequency sensitivity. Thus, prior to starting the experiment, all participants performed an audiometric test over the high-pitched stimuli to ensure their audibility and discriminability (24 tone pairs above 10 kHz, same-different judgment; see details in Supplement). No test was done on participants' ability to discriminate between male and female voices, as this ability is reliably active in humans from the age of 6 months (Miller, 1983). Immediately after the audiometric test, participants were informed that the experiment consisted of a habituation and a test phase. They were instructed that they would hear sequences of sounds in the habituation phase, and that in the tests they would have to rate sequences of sounds as being similar or different from those heard in the habituation phase. We kept the instructions to a minimum, in order to make this work comparable with similar experiments on nonhuman animals. Participants were given the possibility to ask for any clarification on the experimental procedure.

All participants were first habituated to 36 sound sequences following the AB^nA rule. Specifically, each habituation stimulus started and ended with a low tone and contained one to three tones in between (i.e. LHL, LHHL and LHHHL, where L is a tone sampled from the low category and H a tone sampled from the high category). The stimuli design was identical to Ravignani et al. (2013), where each test featured 8 grammatical trials (sound sequences consistent with the AB^nA rule) and 8 ungrammatical trials (evenly distributed between those lacking the first or last low tone, e.g. LHH or HHL). Within the habituation session and each of the test sessions, the order of sound sequences was randomized across participants.

The order of presentation of heterospecific phase and conspecific phase was randomized and balanced across participants. The habituation session was followed by two tests: (i) generalization and extension of the same pattern over novel sound sequences (hereafter "lower abstraction" test), and (ii) generalization over the same structure independent of sound classes, achieved by swapping the high-pitched and low-pitched categories (hereafter "higher abstraction"). In both abstraction tests, half of the stimuli were consistent with the AB^nA structural rule. However, in the higher abstraction test the L-H pattern was inverted, so that As corresponded to high-pitched tones and Bs to low-pitched tones (e.g. HLLLH). Hence, if in the habituation session participants were exposed to an LH*L pattern, in the higher abstraction test phase, they had to classify HL*H patterns as instances of the same AB^nA structural rule as the LH*L pattern. Crucially, no further habituation took place between the two tests. The only difference between lower and higher abstraction tests was given by the acoustic characteristics of the tokens composing the sound sequences (tones vs. syllables). In each trial of both experimental tests, participants listened to one stimulus and were asked to judge whether the sound was "similar to" or "different from" what they heard in the habituation phase. No feedback was provided during the whole experiment.

Ethics statement

The experiment was conducted in accordance with Austrian law and the policies of the University of Vienna. According to the Austrian Universities Act 2002, the appointment of ethics committees is required only for medical universities engaged in clinical tests, the application of new medical methods, and/or applied medical research on human subjects. Accordingly, ethical approval was not required for the present study. Nevertheless, all participants gave written informed consent and were aware that they could withdraw from the experiment at any time without further consequences. All data were stored anonymously.

Audiometry test

As aging might provoke a hearing loss in the area around 11kHz, we wanted to be sure that participants could hear the sound elements our stimuli are composed of. Each trial, the participant

heard two sounds in succession and was asked to judge whether these were the “SAME” or ‘DIFFERENT” sounds.

The audiometric test featured 24 trials, whose order was randomized, divided as follows. 6 trials were pairs of identical, low sounds. 6 trials were two randomly chosen, though different, low sounds. Similarly, 6 trials were pairs of identical, high sounds and 6 more were two randomly chosen, different high sounds. The inter-trial-pause was 500 milliseconds.

Experimental setup

Habituation

The participant heard 3 minutes of habituation stimuli in random order. Even though the length of the stimuli varied, the length of each audio file was normalized to 5 seconds by adding silence.

Tests

Participants were allowed to answer from the stimulus onset onwards (that is, even while a stimulus was playing). Timeout was 7 seconds, counted from stimulus onset. When the trial was over (due to an answer or timeout), the next stimulus was presented after a pause of 2 seconds.

Table A1. Patterns for syllable and pure-tone stimuli. From pg.2 of Ravignani, A., Sonnweber, R. S., Stobbe, N., & Fitch, W. T. (2013). Action at a distance: dependency sensitivity in a New World primate. *Biology Letters*, *9*(6), 20130852.

	habituation	lower abstraction test	higher abstraction test
consistent with the rule	LHL (60), LH2L (120), $LH^{3}L$ (180)	LHL, $LH^{2}L$, LH3L (2), $LH^{4}L$ (2), $LH^{5}L$ (2)	HLH, $HL^{2}H$, $HL^{3}H$ (2), $HL^{4}H$ (2), $HL^{5}H$ (2)
inconsistent with the rule	-	HL, $H^{2}L$, $H^{3}L$, $H^{4}L$, LH, LH^{2}, LH^{3}, LH^{4}	LH, $L^{2}H$, $L^{3}H$, $L^{4}H$, HL, HL^{2}, HL^{3} , HL^{4}
